# Passando da Era de "Verificar o Pulso" para "Verificar o ECG" na Prevenção de Eventos Tromboembólicos na Fibrilação Atrial

**DOI:** 10.36660/abc.20250193

**Published:** 2025-05-28

**Authors:** Tan Chen Wu

**Affiliations:** 1 Faculdade de Medicina da Universidade de São Paulo São Paulo SP Brasil Faculdade de Medicina da Universidade de São Paulo, São Paulo, SP – Brasil; 2 Instituto do Coração do Hospital das Clínicas da Faculdade de Medicina da Universidade de São Paulo São Paulo SP Brasil Instituto do Coração do Hospital das Clínicas da Faculdade de Medicina da Universidade de São Paulo, São Paulo, SP – Brasil

**Keywords:** Fibrilação Atrial, Insuficiência Renal Crônica, Triagem, Diálise, Equipamentos e Provisões

Fibrilação atrial (FA) e doença renal crônica (DRC) compartilham fatores de risco comuns e frequentemente coexistem: 20% dos pacientes com DRC têm FA sintomática, enquanto cerca de 50% dos pacientes com FA terão algum grau de comprometimento renal. Pacientes com ambas as condições têm um risco cinco vezes maior de acidente vascular cerebral, risco três vezes maior de insuficiência cardíaca congestiva, maior morbidade cardiovascular e mortalidade por todas as causas em comparação com pacientes que têm apenas FA ou DRC. As taxas de incidência de FA em um grande banco de dados nacional de Taiwan foram de 5,0, 7,3 e 12,1 eventos por 1000 pacientes-ano na população geral, na coorte de DRC e na coorte de doença renal em estágio terminal, respectivamente. O risco de acidente vascular cerebral ou embolia sistêmica aumentou em 7% para cada declínio de 10 mL/min na eGFR.^1^ A detecção precoce de FA pode reduzir o risco de acidente vascular cerebral e outras complicações relacionadas à FA usando terapia anticoagulante oral ou procedimento de fechamento do apêndice atrial esquerdo.^2,3^

Na primeira pesquisa baseada em médicos conduzida em conjunto pela *European Heart Rhythm Association* (EHRA) e pela *European Renal Association* (ERA)/*European Dialysis and Transplantation Association* (EDTA) em 2020 para obter insights sobre o tratamento da FA em pacientes com DRC, quando questionados sobre o rastreamento de FA entre pacientes com DRC, no geral, 132/295 entrevistados (44,7%) fariam rotineiramente o rastreio da FA em todos os pacientes com DRC em sua primeira apresentação, 68/295 (23,1%) fariam o rastreio para FA apenas em pacientes com DRC selecionados e 95/295 entrevistados (32,2%) não fariam a triagem para FA entre pacientes com DRC. Em comparação com os entrevistados da ERA/EDTA, os entrevistados da EHRA fariam mais frequentemente o rastreio de FA apenas em pacientes com DRC selecionados (28,5% vs. 16,8%, p = 0,017 na primeira apresentação e 36,7% vs. 25,0%, p = 0,031 durante o acompanhamento). Portanto, há uma busca de FA entre pacientes com DRC, mais provavelmente em pacientes com sintomas ou histórico de arritmia. Mais entrevistados da ERA/EDTA fariam o rastreio de FA entre pacientes em diálise ou aqueles com transplante renal funcional em comparação com os entrevistados da EHRA. As técnicas de rastreamento mais comuns foram um único registro de eletrocardiograma (ECG) de 12 derivações (240/288 entrevistados, 83,3%) ou monitoramento com Holter ≥24 h (181/288, 62,8%). Os entrevistados da ERA/EDTA ainda escolheram com mais frequência a palpação do pulso (67,4%), enquanto os entrevistados da EHRA optaram com mais frequência por uma estratégia de monitoramento do ritmo cardíaco usando um dispositivo portátil, telemetria, monitoramento Holter, um monitor cardíaco implantável ou leituras de memória de dispositivo eletrônico cardíaco implantável.^4^

Pacientes com DRC em hemodiálise (HD) representam a "tempestade perfeita" para arritmogênese com uma miríade de fatores contribuintes, incluindo doença cardíaca estrutural subjacente, inflamação, desequilíbrio autonômico, distúrbios eletrolíticos, estresse hemodinâmico e flutuações no equilíbrio hídrico. Tecnologias novas, portáteis e fáceis de usar podem ser uma ferramenta conveniente para triagem de FA, especialmente em populações de alto risco. Nesta edição, Carvalho et al.^5^ avaliaram 388 pacientes (mulheres, 40,7%; idade média de 56,8 anos, DP ± 14,7; tempo de hemodiálise de 27 meses, 10-57) durante uma sessão de HD usando um gadget portátil com uma gravação de derivação única (MyDiagnostick^®^) para pesquisar FA. Cada participante foi rastreado apenas em uma única sessão. A triagem foi positiva em 16 (4,1%) pacientes. A FA foi confirmada por ECG em 7 (1,8%). Sexo masculino (p=0,019), idade avançada (p=0,007), ECG basal alterado (p<0,001), aumento do potássio sérico (p=0,021), redução da pressão arterial sistólica no início da diálise (p=0,007) e angina estável (0,011) foram associados ao rastreamento positivo. O dispositivo apresentou especificidade de 91,74% (IC95% 86,65% a 96,91%) e sensibilidade de 100% (IC95% 100% a 100%), com valor preditivo negativo de 100% (IC95% 100% a 100%) para rastreamento de FA. Os autores concluíram que o uso deste dispositivo se mostrou prático, com alta sensibilidade e excelente valor preditivo negativo. A FA subclínica tem alta prevalência, quase 3 vezes maior que a da população geral, e pode estar subestimada nesta população.^5^

Como limitação do estudo, a prevalência real pode ser subestimada devido à triagem durante uma única sessão de hemodiálise, que ocorre em apenas dois momentos, pois a FA pode se apresentar de forma paroxística, diferentemente da vigilância com tecnologia de monitoramento contínuo. Usando monitoramento de gravador de loop implantável, Koplan et al. detectaram nova FA em aproximadamente um terço dos pacientes com DRC em hemodiálise (18 de 59 indivíduos) em 6 meses. Comparando esses achados com dados anteriores que avaliaram a incidência de FA em uma população geral com função renal amplamente normal em faixas etárias semelhantes (o estudo de Rotterdam), a incidência de FA nesta coorte de pacientes com DRC em diálise foi mais de 10 vezes maior. Entre os pacientes com FA > 6 minutos, 19 de 23 (83%) tinham uma pontuação CHA2DS2-VASc ≥ 2 ou com riscos substancialmente aumentados de acidente vascular cerebral.^6,7^

Da mesma forma, o estudo francês de 71 pacientes com DRC submetidos à HD, dos quais 12 (17%) tinham sido previamente diagnosticados com FA ou flutter. Ao usar ILR, a prevalência geral de FA foi de 37%, e FA de novo foi detectada em 20% durante um acompanhamento médio de 21 meses.^8^ Da mesma forma, um estudo australiano de 50 pacientes com KF em HD encontrou FA paroxística de início recente em 28% dos pacientes durante um acompanhamento médio de 18 meses.^9^

Na era da medicina preventiva, os testes no ponto de atendimento, utilizando tecnologias e ferramentas acessíveis, para rastrear e prevenir complicações previsíveis, recebem interesse significativo para várias aplicações e são certamente bem-vindos.^10,11^ Com a tecnologia vestível se tornando cada vez mais disponível, o potencial para reduzir o acidente vascular cerebral e a mortalidade nessa população vulnerável pode nos permitir fazer a transição de "verifique sempre seu pulso" para "verifique sempre seu ECG" em um futuro muito próximo.
