# Supplementary material for: Passando da Era de "Verificar o Pulso" para "Verificar o ECG" na Prevenção de Eventos Tromboembólicos na Fibrilação Atrial
Source: Arq Bras Cardiol. 2025 May 28;122(3):e20250193. [Article in Portuguese] doi: 10.36660/abc.20250193 (PMC12212375; doi:10.36660/abc.20250193)
Supplement: Translation

# Translation: Moving from the "Check the Pulse" Era To "Check the ECG" in the Prevention of Thromboembolic Events in Atrial Fibrillation

**DOI:** 10.36660/abc.20250193i

**Authors:** Tan Chen Wu

**Affiliations:** 1 São Paulo SP Brazil Faculdade de Medicina da Universidade de São Paulo, São Paulo, SP – Brazil; 2 São Paulo SP Brazil Instituto do Coração do Hospital das Clínicas da Faculdade de Medicina da Universidade de São Paulo, São Paulo, SP – Brazil

**Keywords:** Atrial Fibrillation, Chronic Renal Insufficiency, Triage, Dialysis, Equipment and Supplies

Atrial fibrillation (AF) and chronic kidney disease (CKD) share common risk factors and often co-exist: 20% of patients with CKD have symptomatic AF, whereas around 50% of patients with AF will have some degree of renal impairment. Patients with both conditions have a five-fold increased risk of stroke, three-fold increased risk of congestive heart failure, higher cardiovascular morbidity, and all-cause mortality compared with patients who only have either AF or CKD. The incidence rates of AF in a large nationwide database from Taiwan were 5.0, 7.3, and 12.1 events per 1000 patient-years in the general population, the CKD cohort, and the end-stage kidney disease cohort, respectively. The risk of stroke or systemic embolismincreased by 7% for every 10 mL/min decline in eGFR.^1^ Early detection of AF may reduce the risk of stroke and other AF-related complications using oral anticoagulant therapy or left atrial appendage closure procedure.^2.3^

In the first physician-based survey jointly conducted by European Heart Rhythm Association (EHRA) and European Renal Association (ERA)/European Dialysis and Transplantation Association (EDTA) in 2020 to gain insight into the management of AF in patients with CKD, when asked about screening for AF among CKD patient, overall, 132/295 respondents (44.7%) would routinely screen for the presence of AF in all CKD patients at their first presentation, 68/295 (23.1%) would screen for AF only in selected CKD patients, and 95/295 respondents (32.2%) would not screen for AF among CKD patients. Compared with ERA/EDTA respondents, EHRA respondents would more frequently screen for AF only in selected CKD patients (28.5% vs. 16.8%, p = 0.017 at their first presentation and 36.7% vs. 25.0%, p = 0.031 during follow-up). Therefore, there is a low utilization of screening for AF among CKD patients, most likely in patients with symptoms or a history of arrhythmia. More ERA/EDTA respondents would screen for AF among patients on dialysis or those with a functioning kidney transplant compared with EHRA respondents. The most common screening techniques were a single 12-lead electrocardiogram (ECG) recording (240/288 respondents, 83.3%) or ≥24-h Holter monitoring (181/288, 62.8%). The ERA/EDTA respondents still more frequently chose pulse palpation (67.4%), whereas the EHRA respondents more frequently opted for a cardiac rhythm monitoring strategy using a handheld device, telemetry, Holter monitoring, an implantable cardiac monitor, or cardiac implantable electronic device memory readings.^4^

Patients with CKD on hemodialysis (HD) represent the "perfect storm" for arrhythmogenesis with a myriad of contributing factors, including underlying structural heart disease, inflammation, autonomic imbalance, electrolyte disorders, hemodynamic stress, and fluid balance fluctuations. Novel, portable, and easy-to-use technologies can be a convenient tool for AF screening, especially in high-risk populations. In this issue, Carvalho et al.^5^ evaluated 388 patients (female, 40.7%; mean age 56.8 years old, SD ± 14.7; hemodialysis time 27 months, 10-57) during a HD session using a portable gadget with a single-lead recording (MyDiagnostick^®^) to search AF. Each participant was only tracked in a single session. Screening was positive in 16 (4.1%) patients. AF was confirmed by ECG in 7 (1.8%). Male sex (p=0.019), older age (p=0.007), altered baseline ECG (p<0.001), increased serum potassium (p=0.021), reduced systolic blood pressure at the start of dialysis (p=0.007) and stable angina (0.011) were associated with positive screening. The device presented a specificity of 91.74% (95% CI 86.65% to 96.91%) and sensitivity of 100% (95% CI 100% to 100%), with a negative predictive value of 100% (95% CI 100% to 100%) for AF screening. The authors concluded that the use of this device proved to be practical, with high sensitivity and excellent negative predictive value. Subclinical AF has a high prevalence, almost 3-fold higher than that of the general population, and may be underestimated in this population.^5^

As a limitation of the study, the actual prevalence may be underestimated due to screening during a single hemodialysis session, which occurs in just two moments, as AF can present in a paroxysmal form, unlike surveillance with continuous monitoring technology. Using implantable loop recorder monitoring, Koplan et al. detected new AF in approximately one-third of patients with CKD on hemodialysis (18 of 59 subjects) in 6 months. Comparing these findings with previous data evaluating the incidence of AF in a general population with largely normal kidney function at similar age ranges (the Rotterdam study), the incidence of AF in this cohort of patients with CKD on dialysis was more than 10-fold higher. Among patients with AF >6 minutes, 19 of 23 (83%) had a CHA2DS2-VASc score ≥2 or with substantially increased risks of stroke.^6,7^

Likewise, the French study of 71 patients with CKD undergoing HD, of whom 12 (17%) had previously been diagnosed with AF or flutter. When using ILR, the overall AF prevalence was 37%, and de novo AF was detected in 20% during a mean follow-up of 21 months.^8^ Similarly, an Australian study of 50 patients with KF on HD found new-onset paroxysmal AF in 28% of patients over a mean follow-up of 18 months.^9^

In the era of preventive medicine, point-of-care testing, utilizing accessible technologies and tools, to screen and prevent predictable complications, receives significant interest for various applications and is certainly welcome.^10,11^ With wearable technology becoming increasingly available, the potential to reduce stroke and mortality in this vulnerable population may enable us to transition from "check your pulse always" to "check your ECG always" in the very near future.
